# The Evolved Psychology of Psychedelic Set and Setting: Inferences Regarding the Roles of Shamanism and Entheogenic Ecopsychology

**DOI:** 10.3389/fphar.2021.619890

**Published:** 2021-02-23

**Authors:** Michael James Winkelman

**Affiliations:** School of Human Evolution and Social Change, Arizona State University, Tempe, AZ, United States

**Keywords:** extra-pharmacological effects, evolutionary psychology, innate modules, hominin evolution, ecopsychology, shamanism, neurophenomenology, psilocybin

## Abstract

This review illustrates the relevance of shamanism and its evolution under effects of psilocybin as a framework for identifying evolved aspects of psychedelic set and setting. Effects of 5HT2 psychedelics on serotonin, stress adaptation, visual systems and personality illustrate adaptive mechanisms through which psychedelics could have enhanced hominin evolution as an environmental factor influencing selection for features of our evolved psychology. Evolutionary psychology perspectives on ritual, shamanism and psychedelics provides bases for inferences regarding psychedelics’ likely roles in hominin evolution as exogenous neurotransmitter sources through their effects in selection for innate dispositions for psychedelic set and setting. Psychedelics stimulate ancient brain structures and innate modular thought modules, especially self-awareness, other awareness, “mind reading,” spatial and visual intelligences. The integration of these innate modules are also core features of shamanism. Cross-cultural research illustrates shamanism is an empirical phenomenon of foraging societies, with its ancient basis in collective hominid displays, ritual alterations of consciousness, and endogenous healing responses. Shamanic practices employed psychedelics and manipulated extrapharmacological effects through stimulation of serotonin and dopamine systems and augmenting processes of the reptilian and paleomammalian brains. Differences between chimpanzee maximal displays and shamanic rituals reveal a zone of proximal development in hominin evolution. The evolution of the mimetic capacity for enactment, dance, music, and imitation provided central capacities underlying shamanic performances. Other chimp-human differences in ritualized behaviors are directly related to psychedelic effects and their integration of innate modular thought processes. Psychedelics and other ritual alterations of consciousness stimulate these and other innate responses such as soul flight and death-and-rebirth experiences. These findings provided bases for making inferences regarding foundations of our evolved set, setting and psychology. Shamanic setting is eminently communal with singing, drumming, dancing and dramatic displays. Innate modular thought structures are prominent features of the set of shamanism, exemplified in animism, animal identities, perceptions of spirits, and psychological incorporation of spirit others. A shamanic-informed psychedelic therapy includes: a preparatory set with practices such as sexual abstinence, fasting and dream incubation; a set derived from innate modular cognitive capacities and their integration expressed in a relational animistic worldview; a focus on internal imagery manifesting a presentational intelligence; and spirit relations involving incorporation of animals as personal powers. Psychedelic research and treatment can adopt this shamanic biogenetic paradigm to optimize set, setting and ritual frameworks to enhance psychedelic effects.

## Introduction

Set normally refers to factors related to the person, idiosyncratic personality dynamics, mood and expectations that influence individual experience. Setting is concerned with the social environment, including the broader cultural beliefs regarding the substances and their effects, which contribute to the experience. Set and setting have been considered personal and social influences, respectively, and like ritual, are generally considered to be relatively arbitrary, individual and cultural.

However, concepts of ritual, set and setting must include our evolved dispositions that shape mood, personality, expectations and experiences under the effects of psychedelics. Similarly, setting features include not just cultural features, but also the innate factors which contribute to certain kinds of experiences through the neurophenomenological dynamics produced by psychedelics (i.e., spirits, animal identities). This neurophenomenological experiential dynamics is embodied in the concept of entheogens, where these substances are experienced as producing encounters with internal and external spiritual entities.

There are features of optimal psychedelic ritual, set and setting derived from of our evolved psychology. For example, most prefer psychedelic sessions at night and would feel it strange to start a ceremony at midday. Singing, drumming and dancing are frequent aspects of psychedelic rituals, but not wrestling, swimming or mountain climbing. Feeling compelled to sing a song or heal is normal in psychedelic sessions but explaining a mathematical solution or football strategy is not. Why?

The natural aspects of set and setting reflect an evolutionary relationship between our evolved psychology and the effects of psychedelics as exogenous analogues of neurotransmitters. Consumption of mind-altering plants enhanced human adaptations across evolution (Sullivan et al., 2008). Evolutionary, ecological and ethology perspectives reveal a multi-million-year relationship of humans with psilocybin-containing fungi. Regionally specific psilocybin-containing fungi are found in most ecozones (Guzmán et al., 1998), indicating their presence as environmental influences that affected hominin populations in all the major habitable regions for millions of years. Deliberately seeking and consuming plants for their bioactive properties is a hominid trait attested to in wild chimpanzees’ consumption of dozens of plants without nutritional value but with medicinal properties (Krief et al., 2005; Forbey et al., 2009; Masi et al., 2012). Chimpanzees’ intentional self-medication for diseases, wounds, and bacterial infections indicates ancient hominins (uniquely human ancestors) also practiced deliberate self-medication. Given humans uniqueness in deliberate uses of psychoactive plants, hominin evolution must have expanded these abilities, and use of psychedelics is a clear example.

Psychedelics shaped human evolution as a significant source of serotonin analogues that enhanced active stress response mechanisms. The 5 HT2 psychedelics (those that bind at the serotonin receptors) stimulate 5-HT2AR, a serotonin receptor characterized by enhanced plasticity that enables an active coping strategy that addresses stress sources through changes in perspective and behavior (Carhart-Harris and Nutt, 2017; also see below), contributing to selection for qualities of human nature and our evolved psychology. Use of psilocybin as exogenous neurotransmitter analogues was eventually integrated within collective rituals, which played an important role in adaptations that facilitated individual and collective well-being. This pre-modern use of psychedelics involved shamanism, a cross-cultural pattern of ritual practices and beliefs that reflects aspects of our evolved psychology (Winkelman, 2010a; Winkelman, 2015a). Shamanism engaged activation of innate cognitive processes and endogenous healing responses, which are also effects of psychedelics.

The deep evolutionary roots of collective rituals reveal a psychosocial dynamic that has characterized hominins for millions of years (Winkelman, 2009; Winkelman, 2010a; Winkelman, 2010b). The co-evolution of shamanism and ritual ingestion of psilocybin-containing mushrooms shaped our evolved psychology (Winkelman, 2013a), and consequently the features that optimally access those set and setting for entheogenic experience. Therefore, the principles of shamanism provide important guidelines for optimizing the modern applications of psychedelics to our evolved psychology.

But there is little recognition in pharmacology, psychiatry or ethnopharmacology of the significance of millions of years of enabling relationships of psilocybin-containing mushrooms with humans’ evolved psychology of ritual healing (but see Winkelman, 2010a; Winkelman, 2013a; Winkelman, 2014; Winkelman, 2019b). These relationships imply an evolved set and setting, which is to say that humans acquired specific adaptations that affect an optimal set (psychological orientation) and setting (social and cosmological context) for the effective utilization of the natural properties of psychedelics. These adaptations likely occurred because psychedelics enhanced human social relations. These and other enhancements of social and cognitive processes could have contributed to key evolutionary developments involving cultural niches and shared social beliefs, which mediate our adaptation to the environment (also see section below “The cognitive niche”).

But little literature exists on the nature of the shamanic set and setting that characterized most of humanity’s relationships with psychedelic fungi (but see Winkelman, 2007; Metzner, 1998). This shortcoming is addressed in this interdisciplinary review synthesizing the author’s publications (Winkelman, 1990, Winkelman, 1992, Winkelman, 2002; Winkelman, 2007; Winkelman, 2009; Winkelman, 2010a; Winkelman, 2010b; Winkelman, 2011; Winkelman, 2013a; Winkelman, 2013b; Winkelman, 2014; Winkelman, 2015a; Winkelman, 2015b; Winkelman, 2017a; Winkelman, 2017b; Winkelman, 2018; Winkelman, 2019a; Winkelman, 2019b; Winkelman, 2019c; Winkelman, 2019d; Winkelman, 2019e). These cross-cultural studies of shamanism and evolutionary psychology are integrated with recent findings from clinical and cognitive neuroscience studies that identify adaptative effects of psychedelics on cognition, personal well-being and social relations. This neurologically based practice of shamanism provides orientations for structuring contemporary psychedelic therapy approaches to ritual, set and setting in ways consonant with the psychological, social and cosmological dimensions of our evolved ecopsychology.

## Psychedelics in Evolution: Exogenous Neurotransmitter Sources

There is a deep evolutionary relationship between humans and plant drugs (Sullivan et al., 2008; Sullivan and Hagen, 2015). There were diverse evolutionary influences of plant substances from effects such as: enhanced vigilance and sensory and mental acuity; increased pain management and endurance; enhanced mating opportunities; and reduction of stress, defensiveness and depression. Exogenous analogs of human neurotransmitters found in plants effect many neurotransmitter systems, including serotonin, dopamine, acetylcholine, norepinephrine and dopamine (Passie et al., 2008; Hintzen and Passie, 2010; Ray, 2010).

Human use of psilocybin and other psychedelics provided exogenous analogues for neurotransmitters that are limited by dietary intake. Our ancestors accrued fitness benefits through utilization of exogenous sources of neurotransmitters, with ancient environmental exposures to these exogenous sources of neurotransmitters stimulating human evolution. Hominin access to psychoactive fungi would have been regulated by the highly seasonal dynamics of fungi growth (i.e., the presence of bovines for coprophilous species, as well as rainfall and temperature conditions). Nonetheless, exposure to these and other botanical sources of neurotransmitter analogues was sufficient for hominins to undergo positive selection for CYP2D6 (Cytochrome P450 2D6), a gene that encodes an enzyme that facilitates metabolization of plant toxins (Sullivan et al., 2008), reducing their toxicity and enhancing their bioavailability. Hominins also underwent selection for genes producing precursor molecules for several endogenous opioids and neuropeptides (Rockman et al., 2005). The high affinity of CYP2D6 enzyme for plant alkaloids such as natural psychedelics and other psychoactive drugs (Kennedy, 2014) indicates significant selection pressures were exerted on hominin populations by environmental sources of secondary metabolites that affected the evolution of the human nervous system.

Substances with effects on serotonin are important in the context of human evolution because “serotonin neurotransmission enhances two distinct adaptive responses to adversity, mediated in large part by its two most prevalent and researched brain receptors: the 5-HT1A and 5-HT2A receptors. . . Active coping (i.e. actively addressing a source of stress) is mediated by 5-HT2AR signaling and characterized by enhanced plasticity (defined as capacity for change). . . . [This] 5-HT2AR pathway is enhanced by 5-HT2AR-agonist psychedelics” (Carhart-Harris and Nutt, 2017, p. 1091). This differs from the brain’s default response to adversity, a passive coping strategy involving toleration of sources of stress (mediated by postsynaptic 5-HT1AR signaling); instead, psychedelics stimulate an active coping strategy mediated by 5-HT2AR signaling.

This evolution of the hominin serotonergic system involving modifications of 5-HT2AR signaling enhanced the capacity to actively address sources of stress through changes in perspectives and behavior, rather than passive tolerance of stressors. The ability of psilocybin to functionally modulate 5-HT2A receptor signaling provides enhanced neuroplasticity, a fitness enhancing effect supporting an active coping strategy with heightened flexibility in both unlearning and learning, particularly creating new models of adaptation.

How do psychedelics enhance the creation of new perspectives and behavior and what is the basis of the new perspectives they produce?

### Serotonergic Regulation and Psychedelic Deregulation

The role of serotonin in the inhibition of dopamine is central to neurochemical balance in the brain, involving the right hemisphere’s serotonergic and noradrenergic systems inhibiting the dopamine system and the left hemisphere (Previc, 2009). Serotonin is the primary neurotransmitter system affected by psychedelics, especially through agonism of 5-HT2A receptors (Nichols, 2016). In addition to activation of serotonin receptors, psilocybin and psilocin also stimulate dopamine receptors (Ray, 2010). In addition to their stimulatory effects, 5HT2 psychedelics also have blocking (antagonist) effects at some serotonin receptors. Psychedelics exbibit phasic effects, first stimulating and enhancing serotonergic activity; secondly, saturating and locking out the serotonin receptors; and thirdly, consequently releasing the habitual serotonin repression of the dopaminergic system (Passie et al., 2008; Hintzen and Passie, 2010).

Psychedelics lock into serotonin receptors but are resistant to normal serotonin reuptake mechanisms that remove neurotransmitters from the receptor to increase synaptic firing frequency. This psychedelic resistance causes habituation of receptor firing, and eventually reduces the regulatory role of the serotonergic system because receptor sites become locked out. The resulting loss of serotonin control results in a release of the dopamine system normally repressed by serotonin, causing a variety of visual experiences (hallucinations, dreams, psychosis) and modifying control of major brain subsystems.

These psychedelic effects in altering consciousness are illustrated by Vollenweider’s (2001) findings on the mechanisms of action of psychedelics on the frontal-subcortical circuits, a principal organizational network of the brain uniting cortical areas with the brain stem region. Psychedelic resistance to reuptake results in the impediment of habitual serotonergic suppression of the ascending flow of information. These effects are typified by psychedelics’ interruption of cortico-striato-thalamo-cortical loops that inhibit the lower brain structures’ sensory gating systems, consequently releasing a flood of information from ancient brain processes normally inhibited by serotonin (Vollenweider and Geyer, 2001). The psychedelics’ effects on the cortex are not undifferentiated but involve a localized decrease in thalamus information gating and increased bottom-up connectivity from the thalamus to the posterior cingulate cortex and the ventral striatum (Preller et al., 2019). Preller et al. (2019) confirm this CSTC model of psychedelic action in disintegration of normal information processing within these loops, accompanied by increases in effective connectivity in the CSTC pathways involved in sensory and sensorimotor gating of information transmitted to the cortex. These effects are mediated by 5HT2 increases in effective connectivity of the thalamus with the posterior cingulate cortex.

This enhanced availability of information from ancient brain areas normally repressed results in a flood of information that overwhelms the frontal cortex, altering experience of self, others, and environment and promoting a focus on the internal world of psychological projections. Psychedelics also stimulate the visual system and limbic system areas that manage emotional information and mediate personal relations and social bonding as well as the range of innate brain modules. In addition to the 5HT2 psilocybin and psilocin effects on serotonin receptors, they also have effects on dopamine receptors (Ray, 2010). Psilocybin increases striatal dopamine concentrations (Vollenweider et al., 1999), an area of the brain mediating rewards from social interactions.

### Innate Intelligences in Psychedelics and Shamanism

The effects of the deregulation of these brain areas caused by serotonin saturation and dopamine disinhibition have direct effects on experience, manifested in the release of normally unconscious sensory, personal and emotional dynamics. The bottom-up brain dynamic induced by psychedelics liberates aspects of the unconscious mind. Phenomenal evidence indicates that the brain’s innate intelligences, modular structures and cognitive operators are liberated by psychedelics, with their integration producing supernatural thought and experiences (Winkelman, 2017a; Winkelman, 2018).

Psychedelic stimulation of innate brain structures was discovered a century ago in mescaline-induced visual phenomena called entoptics. Subjective reports of mescaline-induced experiences revealed recurring visual patterns, form constants such as geometric shapes, lattice structures, tunnels, funnels, and cones; and their super-imposition and integration into larger complex flowing patterns that typify psychedelic experiences. The release of these and other innate cognitive aspects by psychedelics make these cognitive structures part of the intrinsic features of set and setting, a manifestation of the structures of the human brain.

Evolutionary psychology proposes human cognitive evolution involved acquisition of independent modular cognitive structures providing specialized innate capacities for specific survival functions, automatic processors operating unconsciously to provide specific cognitive responses that affected adaptation in the environment of evolutionary adaptedness (Barkow et al., 1992; Carruthers and Chamberlain, 2000; Barkow et al., 2001). These innate intelligences manifest in unconscious functions operating through functionally specialized modular adaptations. These innate intelligences or operators (d’Aquili and Newberg, 1999; Ernandes, 2013) were acquired as ancient adaptations affecting survival, such as detecting an agent, recognizing animal species, inferring the thoughts of others, and imitation and interpretation of behaviors. Gardner (1983, 2000) proposed ten innate human intelligences, biopsychosocial potentials of humans manifested across cultures (see Table 1); Winkelman (2017a, 2018, 2019d) has shown these are the basis for psychedelic phenomenology, supernatural thought and shamanism.

**TABLE 1:** Gardner’s innate intelligences and supernatural concepts.

Innate intelligences	Component of supernatural experiences
Bodily-kinesthetic (mimesis)	Ritual enactment (imitative magic)
Intrapersonal—One’s own mind capacities	Spirits and souls
Interpersonal (social)—a “theory of mind”	Spirit communication, divination
Language/symbolism	Unseen reality, divinatory meanings
Logical-mathematical	Form constants (entopic phenomena)
Musical	Produce endorphin and opioid responses
Spatial	Out-of-body experience
Naturalist, animal classification	Animal identities and powers
Spiritual, noetic and transcendent experiences	Spirit beliefs, Animism
Existential intelligence, cosmic explanations	Mythology, pantheons

### Entheogenic Experience as a Psychedelic Neurophenomenology

Psychedelics produce animism, an entheogenic mind set where the natural world is humanized and personalized with traits (sentience, relationality) that derive from humans’ innate social and cognitive intelligences (Winkelman, 2013a). Psychedelic experiences exhibit phenomenological features corresponding to Gardner’s innate intelligence (Winkelman, 2018). These social and cognitive modules are exemplified in contemporary entheogenic experiences of communication from psychedelic beings reported as entities, gnomes, dwarfs, elves, imps, goblins, “little people,” and human-like angels, spirits, and gods.

Cognitive science explanations of religion (Pyysiäinen, 2009; Boyer, 2017; Clements, 2017; Winkelman, 2019d) also propose universal features of religious thought reflect the operation of innate modular structures. Key features of spirits reflect the combined operation of innate operators for self awareness (intrapersonal intelligence) and other representation (interpersonal intelligence), a capacity for “mind-reading” (inference of others’ thoughts) that are evolved mechanisms for adaptation to the central factor affecting human survival—actors in the social environment.

These combinations of innate cognitive capacities is how supernatural experiences and beliefs contributed to new forms of intelligence, creating symbols from the integration of operations from distinct cognitive modules that normally operate independently (Winkelman, 2002; Winkelman, 2010a; Winkelman, 2019d). ASC have a functional effect in producing such integrated synesthesia experiences and symbols (Winkelman, 2002), with the combination and integration of normally separate operational systems provoked by diverse ASC (Winkelman, 2011). The combination of innate modular functions in producing supernatural thought is exemplified in animism, where the natural world is attributed properties of humans’ own self-awareness, cognition and emotions; and conversely, in shamanic features of animal powers involving linking of the naturalist (animal species) module with self awareness and social identity.

#### Animism and Spirits as Innate Intelligences

Animism, a view that that nature involves sentient entities that interact with humans, is fundamental to shamanism and entheogenic perspectives. Animism reflects operations of innate processing modules for “animacy detection,” for being hyper-sensitive to the presence of an animate agent. The religious presumption of an unseen agent, a hyperactive sensitivity and automatic tendency to project an active agent responsible for the cause of unexplained phenomena, reflects functions acquired for detection of predators and prey. These tendencies were expanded across human evolution because of survival benefits of detecting predators, and subsequently linked to other capacities to understand the most important and dangerous animals in the environment—other humans. Consequently, our animistic thinking also emphasizes human-like mental, personal and social qualities.

Spirit beliefs extend animism and agency detection with Gardner’s intrapersonal and interpersonal intelligences, integrating capacities for self-awareness and mind-reading. Intrapersonal intelligence provides a meta-cognitive operator that uses its capacity for self-awareness and representing one’s own mental states to model and infer the contents of others’ minds. The need for human cooperation requires knowledge of their mental states, a “theory of mind” that infers others’ intentions (goals) and beliefs. Our tendency to attribute our mind states, internal dispositions and purpose to spirit entities reflects this adaptive tendency to infer the mental states of others to predict and adapt to their behavior. Our evolved psychology for modeling the cognitive and emotional world of others also produces the imagined entities of the spirit world, extending social adaptations (Weingarten and Chisholm, 2009; also see; Rossano, 2006; Winkelman, 2007).

A fundamental human capacity for socialization involves process for modeling self development based on internalization of others’ roles. This process can be extended to internalization of spirit others as personal models for development. This process is also extended in the use of natural symbols (animals) for representations of self and social groups (totemism).

#### Animals as Innate Intelligence and Metaphor

Our naturalist (animal) intelligence provides for recognition and classification of animal species in similar systems found in cultures worldwide that parallel the scientific Linnean classification system. Humans in different cultures parse the natural world of animal species similarly through our innate intelligence. This animal intelligence provided templates for analogical reasoning, for creating meaning in animal metaphors. This intuitive biology was subsequently extended as a natural system for creating personal identity (animal powers) and social identity (totemism).

A prominent feature of shamanism involves animal allies that takes the social capacity to incorporate the models of the social other—to internalize qualities of others into our self-identity—and extends this capacity by using animals as representations of self. The characteristics of animals are used to structure individual psychodynamics and social behavior modeled on the natural symbols of animals. A similar model of identity is exemplified in totemism, where an animal species represents the identity of a group, as manifested in totemic ancestor worship where group deities are represented by an animal species. The significance of totemism as symbolic cognition was illustrated in Levi-Strauss (1962) book *Totemism*. Totemism involves a metaphoric relationship between the natural history domains of animals and domain of social groups, conceptualizing humans’ social organization through analogical reasoning processes that attribute a homology between animal species and human groups.

### Visions as Innate Symbolism

Psychedelic disinhibition of the dopaminergic system through reduction of serotonergic control results in ascendance of the dopaminergic systems which produce a variety of visual syndromes, typified by hallucinations and dreaming (Hobson, 2001). The information-rich visual experiences produced by psychedelics reflect increased visual cortex activity from activation of multiple serotonin (5-HT) receptors and networks and increases in cortical excitability (Kometer et al., 2015). Such visual images are also induced by hyperactivation of dopamine receptors and the blockage of glutamate receptors (Rolland et al., 2014), showing that they are an inherent potential of human nature, rather than just a product of a specific neurotransmitter effects.

Psychedelics increase blood flow in the visual cerebral cortex and expand functional connectivity in the primary visual cortex, reflecting an enhanced activity in visual areas from increase in contributions from other areas of the brain to the visual processing centers (Carhart-Harris et al., 2016; Preller et al., 2019). Psychedelics increase connectivity within brain regions responsible for vision, producing a more unified brain, with connections between disparate regions that normally lack communication with each other. Psychedelics (LSD) enhance primary processing functions (Kraehenmann et al., 2017) involving effects on 5-HT2A and 5-HT1A receptors that stimulate subcortical and limbic areas, increasing availability of unconscious mental processes.

#### Presentational Symbolism: A Visual Epistemology

These forms of thought presented in psychedelic experiences involve what Horváth et al. (2017) call visionary phantasy, a polysemic and multimodal manifestation involving images, affective responses, imagination, and significant personal and intellectual realizations. This visionary phantasy induced by psychedelics is primarily visual, but also includes corporeal and sensory experiences, affect, and diverse forms of ideation. These experiences induced by psychedelic stimulation of innate aspects of our visual system is also manifested in: dreaming, fantasy and day-dreaming; hypnagogic and hypnopompic imagery; out-of-body and near-death experiences; hallucinations associated with toxic exposures, illness and disease; and shamanic and mystical experiences.

Psychedelic-induced visionary phantasy reflects a latent human cognitive capacity underlying all experience that manifests through the visual system used for organizing information in the external world. Carhart-Harris et al. (2016) found LSD induced functional profiles and cerebral response are similar to viewing the world with eyes open in normal waking consciousness. Ayahuasca-induced visual effects in the primary that visual centers involve activation of brain areas similar to those produced with the eyes open (de Araujo et al., 2012).

These internally produced visual experiences evoked by diverse conditions are symbols—presentational symbolism—an ancient modality of cognition (see Winkelman, 2010a Chapter 3 for review). This visual cognition is a natural system that emerges from unconscious brain processes, exemplified in dreams, and manifesting spontaneously and involuntarily with interference in the habitual repressive regulation of the visual cortex. This ancient mode of imaginal consciousness also appears in dreams, which use this visual presentation for rehearsal, integration of learning and problem solving. This capacity for meaning provides knowing directly in a form that preceded language-based consciousness. These visual dynamics underlie the affective cognition system that functions constantly in our daily life, presenting material from deep personal affective layers of consciousness. This form of thought involves image schemas derived from sensorimotor experience and learned structures that represent our inner mental life and thought and relationships with the external world (Winkelman, 2010a; Winkelman, 2015a).

Academia has recognized this intuitive mode of knowing for centuries, but it is seldom studied because of difficulty in sharing these internal experiences. This illusive capacity is reliably elicited by psychedelics, which are unparalleled tools for examination of the phenomenology and operation of this intrinsic system of the human brain-mind and a source of unconscious cognitive processes. This visual thinking capability involves the innate intelligence of spatial-temporal reasoning, an ability to think though visualizing patterns and performing mental manipulations with them. The visual thinking provides the working space for organizing constructs, assimilating information and creating new ideas by synthesizing spatial information. This capacity was central to human evolution.

### The Cognitive Niche

A cognitive niche is how we adapt to diverse environments, a uniquely human capacity involving reliance upon complex cognitive systems of representation that mediate relations with the natural world (Boyd et al., 2011; Whiten and Erdal, 2012; Laland et al., 2016). This revolutionary change produced the uniquely human ability to live within shared symbolic representations encoded in cultural models of the world. The cognitive niche is a social mind that derives from humans’ highly cooperative communicative and symbol-making consciousness, an information-rich, culturally constructed environment that defines the niche, the culturally relevant aspects of the physical environment (Odling-Smee et al., 2003). Traditional cultural orientations mediated these relations with the natural world through cognitive systems called myths and cosmologies.

The model of psychedelic instrumentalization derived from the drug instrumentalization paradigm (Müller and Schumann, 2011) indicates hominins experienced selective advantages from the contributions of psychedelics to enhancement of the niche–construction processes and the socio-cognitive niche. Psychedelic-induced 5HT2A active stress responses involving modification in perspectives and/or behavior (Carhart-Harris and Nutt, 2017) would have affected niche construction through enhanced creativity and problem solving. 5-HT2A receptor signaling played an important role in human evolutionary and ontogenetic development through enhancing plasticity and adaptability during extreme conditions (Carhart-Harris and Nutt, 2017).

Early hominins evolved traits from selection pressures for abilities to live in a cognitive niche, a virtual social reality, for functioning and survival. Beginning with the incidental ingestion of psychedelic fungi in an opportunistic diet, and eventually their deliberate inclusion in rituals, our ancestors’ use of psilocybin could have contributed to the evolution of our unique survival mode by imposing a systematic bias on the selective environment via the enhanced visual information processing and integration induced by psychedelics. Psychedelic consumption thus could have had significant consequences on the selective forces that drove hominin cognitive and behavioral evolution.

These adaptations involving construction of new models of the environment were enhanced by psychedelic effects on visual thinking and globally integrated cognition. The enhanced availability of information is a central feature of psychedelic effects, a result of increased global connectivity in the brain that results from psychedelic interference with the integrity of the Default Mode Network (DMN). Disabling the DMN results in increased levels of functional connectivity between normally disconnected brain networks (Roseman et al., 2014) and more communication across the entire brain with very strong links and topologically long-range functional connections and a wider range of connectivity states (Tagliazucchi et al., 2014; Preller et al., 2018). Psilocybin produces a new dynamic of coordinated oscillations across brain regions (Kometer et al., 2015), with overall phase synchronization coordinating EEG across diverse brain areas and producing greater global neural integration (Carhart-Harris et al., 2014).

Psychedelics consequently produce a higher level of brain integration with a greater diversity of functional connectivity networks. This enhanced modeling provided a meta-context for possible further selection of related traits and competencies that facilitated adaptation to the socio-cognitive niche. Individual differences in enhanced cognitive processes provoked by psychedelics provided variations in fitness in ancient hominin populations, variation upon which selection could act because they contributed to enhanced ability to operate in a cognitive niche.

## Shamanism as the Evolved Ritual Context of Psychedelic Use

Ancient hominin societies developed social institutions, represented in the practices of shamanism, to manage the therapeutic and other adaptive effects that can be obtained by using psychedelics to integrate information in consciousness. The deliberate, intentional self-administration of psychoactive compounds that significantly enhance mental functions and access to unconscious material is typified in shamanic practices, which exhibit hominins’ unique capacities for constructing a cognitive niche through adaptively instrumentalizing these mind-altering substances to produce sociality-enhancing models of spirits.

Shamanism represents a cross-cultural concept derived from recognition of a similar complex of spiritual healing practices in pre-modern cultures around the world. Europeans first developed this concept from reports by explorers, traders, missionaries, colonists, administrators and military personnel. Their sensationalistic exaggerations and misunderstandings, filtered through Christian religious biases, were incorporated into the literary and cultural life of elite Renaissance Europe during the 18th century as an understanding of the foreign “other” (Flaherty, 1992). These conceptualized shamanism as representing humans’ irrational nature where charisma and dramatical emotional rituals dominated social life.

This European view of shamans as theatrical performers who deceived their gullible community only began to shift as professional understandings emerged with 19th and 20th century anthropological studies. Studies on Siberian groups already substantially changed by Russian colonization were soon augmented with ethnographies that attested to a similar cross-cultural phenomenon manifested in European, Asian, Austronesian and American indigenous cultures. The modern concept of the shaman, based on the term *saman* in the Tungusic language, took this widespread Siberia cognate and applied it cross-culturally to premodern spiritual healing practices.

The concept of shamanism began its scientific basis in Mircea Eliade’s (1951/1964) *Shamanism: Archaic Techniques of Ecstasy,* where this comparative religion specialist described these recurrent practices found across the globe. This contributed to the 20th-century emergence of the shaman as a legitimate comparative (etic) concept in academia and subsequently its revitalization and popularization in Western society. Eliade characterized the core of shamanism as “techniques of ecstasy”—ritually-induced altered states of consciousness (ASC)—used in community ritual interactions with the spirit world for purposes of healing and divination. This nighttime ceremony attended by all members of the local group was an unparalleled social gathering, a “spectacle unequaled in the world of daily experience” (Eliade, p. 511). This dramatic ritual provoked powerful emotions as the shaman recounted battles with spirits while excitedly beating drum, imitating animals, singing, chanting, and dancing. Eventually the shaman reclined or collapsed exhausted into a ecstatic state “a trance during which his soul is believed to leave his body and ascend to the sky or descend to the underworld” (Eliade, 1951; Eliade, 1964, p. 5) to communicate with the spirits and obtain their cooperation. This soul flight was also experienced as the personal transformation into an animal to use its powers. Soul flight and animal powers were key to the shaman’s activities, which included healing, divination, clairvoyance, acquiring information about group members, hunting, recovery of lost souls, communication with spirits of the dead, escorting souls of the dead, and protection against spirits and sorcerers.

### Cross-Cultural Features of Shamanism

While some have questioned Eliade’s claims that shamanism was cross-cultural because of his loose comparative methods, the broad patterns he noted are confirmed by formal cross-cultural studies (Winkelman, 1990; Winkelman, 1992; Winkelman, 2010a). Foraging societies worldwide have practitioners with the central features Eliade attributed to shamans, including:

- Preeminent leader in the group’s ecological, political, spiritual and healing activities;
- Performance of a communitywide nighttime ritual to engage and enter the spirit world;
- Ritual preparations involving fasting and water deprivation, sexual abstinence and austerities;
- Training and rituals involving alteration of consciousness produced though extensive drumming, dancing, collective chanting and singing, and often use of plant drugs;
- Experiences conceptualized as soul flight, similar to modern astral projection, out-of-body and near-death experiences, in which the shaman travels into the spirit world;
- Ritual healing of soul loss, the extraction of intrusive objects, and sorcery;
- Selection from encounters with spirits in visions, illness, and dreams;
- Training involving prolonged solitude in the wilderness, a vision quest for spirit allies that empowered the shaman;
- An initiatory experience of personal death, often involving experiences of being attacked, killed, and devoured by animals;
- A rebirth experience in which the animals reconstruct and revive the initiate by incorporating their powers into the initiate;
- Experience of a personal transformation into an animal; and
- Causing magical harm through intrusive darts and soul theft.

This complex of ritual activities and beliefs found worldwide in foraging societies establishes shamanism as an empirical reality of the premodern world, not something created by the Western imagination. The cross-cultural distribution of the shamanic features reflects a cultural universal: all societies have ritual practices involving alterations of consciousness for spirit communication, divination and healing, what Winkelman (1990) called shamanistic healers. These cultural universals of ritual ASC, spirit engagement, divination and healing reflect intrinsic aspects of human nature involving innate intelligences (Winkelman, 2019d).

### Biogenetic Origins of Shamanism in Hominid Displays

Biogenetic structural approaches reveal shamanism has roots in hominid communal displays (Winkelman, 2009; Winkelman, 2010a; Winkelman, 2010b; Winkelman, 2015a; Winkelman, 2019c). Collective ritualized behaviors provided the most significant social institutions of hominids and early modern human societies (Winkelman, 2002; Rossano, 2006; Rossano, 2007; Rossano, 2009; Winkelman, 2009; Rossano, 2011; Rossano, 2015). This reflects the social importance of collective ritualization in primates in general, and the expansion of these capacities for communication and social coordination during hominin evolution (Winkelman, 2015a). Shamanic rituals have evolutionary roots in these collective reunification displays manifested in the chimpanzees’ maximal display (Winkelman, 2009; Winkelman, 2010b).

These origins of human ritual are identified in these comparative analyses revealing parallels of shamanism with the chimpanzee maximal display. This reveals a generalized hominid trait involving bipedal displays by the alpha male and collective hand and foot drumming and chorusing by the group (Lawick-Goodall, 1968; Lawick-Goodall, 1971; Goodall, 1986). The maximal display extends the bipedal charge with beating on logs and trunks, hurling rocks and branches, and leaping and beating the ground, accompanied by screaming and hooting which may escalate into a physical attack on group members lacking submissive behaviors.

This maximal display is the basic mechanism for group integration in chimpanzee society by producing an auditory beacon to reunite the dispersed troop (De Waal, 1997). These nightly performances integrate dispersed members, illustrating how collective rituals enhance group integration and survival. These common behaviors of great apes (Lawick-Goodall, 1968; Geissmann, 2000) reflect ancient hominid dispositions for overnight collective ritual displays with collective vocalizations. These provided functions of group integration through communicating group location, moderating emotional states, and enhancing group cohesion (Geissmann, 2000; Merker, 2009).

This common dynamic of great apes indicates these behaviors characterized our hominid ancestors and consequently, our uniquely human ancestors, the hominins. These pre-adaptations for hominin ritual include:

- nighttime group reunification displays;
- aggressive alpha-male enactments (“dancing”);
- emotional group vocalizations (chorusing and singing) and drumming; and
- individual and group emotional integration.

Synchronous group vocalizations are central to both hominid displays and shamans’ rituals, an expressive system for communicating emotional states and enhancing group integration. Their functional adaptations include (Winkelman, 2010a): an auditory beacon facilitating group re-integration for protection; intergroup boundary maintenance; a signal of social hierarchy, reducing intragroup conflict and violence; an intimidating costly display that deters predators; and a release of tension producing group emotional synchrony.

### Hominid Displays and Shamanic Ritual: The Evolutionary Gap

The similarities between chimpanzee maximal displays and shamanic rituals indicate a hominid biogenetic behavior pattern which was a pre-adaptation for shamanism. This involved collective nighttime group vocalizations and drumming with vigorous attack displays by alpha males (Winkelman, 2009; Winkelman, 2010a). The features of shamanism that emerged over hominin evolution are revealed by the differences between this hominid baseline and shamanic rituals. These differences, involve:

- evolution of capacities for extended drumming, dancing, song and music;
- expansion of the ritual capacities for more prolonged conspicuous displays;
- rituals of healing;
- beliefs regarding spirits, particularly animals as personal powers and identity;
- prominent alterations of consciousness and their cognitive properties;
- diagnosis and divination of information;
- mythic systems of explanation.

Dunbar (2017) proposed shamanic practices originated in community bonding rituals which evolved as adaptations that elicit endorphin responses to enhance social bonding. The enhancement of bonding mechanisms in hominin evolution extended the primarily dyadic relationships characteristic of chimpanzees to bonding among all members of the group characteristic of shamanic rituals. Music and synchronous movement such as dancing provokes the release of endogenous opioids and stimulates neurotransmitter systems (dopamine, norepinephrine) (Launay et al., 2016; Tarr et al., 2016). Communal singing elicits oxytocin production (Panksepp and Trevarthen, 2009; Chanda and Levitin, 2013), a neurohormone enhancing social bonding. Music, chanting and rhythmic activities such as drumming, dancing and clapping elicit endorphin responses that extended the group capacity for social bonding (Dunbar, 2014). Shamanism exemplifies the human capacity for group production of synchronized clapping and dancing and harmonized song and music for prolonged periods. These group enhancement technologies exploited an innate operator that Gardner called a bodily-kinesthetic intelligence involving mimesis that functions through the operation of mirror neurons (see Garrels, 2005; Garrels, 2011; Winkelman, 2015a for review in relationship to shamanism).

### The Roles of Mimesis in Evolution

The capacities of music and dance depend on mimesis, an innate modular intelligence and a central feature of hominin evolution which emerged 1–2 million years ago (Donald, 1991). Mimesis is a multimodal capacity that enables the body to entrain its movements with external rhythms, such as exhibited in dance and music. These capacities of mimesis derive from a neurocognitive adaptation involving mirror neurons that supports multiple expressive capacities—mime, gesture, imitation, music, song, dance, and observational learning (Donald, 2006; also see; Garrels, 2011). Donald shows how the mimetic capacity to represent and communicate through enactment provided the basis for an archaic level of culture expressed through imitation, gestures, pantomime and shamanism. Girard (1987) examined the extensive implications of the capacities of mimesis which had primordial roles in the foundations of culture, and eventually religion.

Mimetic processes are based in the activity of mirror neurons, brain cells that are activated by performance of a specific intentional and goal directed behavior, as well as when one observes a conspecific engaging in that same specific intentional movement (see Garrels, 2005; Garrels, 2011 for reviews). Mirror neurons function as both motor and sensory neurons, reflecting a common neural basis for both performance and observation/understanding of behavior. This common basis for action and perception creates a shared experience for actor and observer through neurologically mediated responses of mirror neurons. Mirror neurons mediate social behavior by providing a basis for intuitive understandings of conspecifics, especially though their goal-directed actions. This common capacity for understanding perceptions of a behavior and production of that behavior provides a system of shared consciousness and meaning that was the foundations of human personal and social consciousness long before the development of spoken language. Mimetic expressions are understood at intuitive and nonconscious levels, providing fundamental interpersonal communication processes.

#### The Functions of Mimesis

Mimesis is an adaptation providing an innate capacity for communication through the body, a pre-language expressive system of early hominins involving the ability to intentionally represent through imitation, gestures, pantomime and emotional expressions (Donald, 1991; Donald, 2006). While mimesis operates at pre-conscious levels of intention and awareness, imitation extends mimesis in a more conscious and deliberate copying of an act for social communication (Girard, 1987). Mimesis has a threefold sequence and functions—imitation, representation and construction—which provides the basis for mediation between inner impressions and the experiences of the exterior world, the bases for the construction of a perceived external reality (Klein, 2003). This construction of the “inner world” is a symbolic world based on behavior, the actions that produce experiences, which are used for the symbolic constructions of reality. The experiences of both internal and external realities are based in memories of the impulses produced by the body that generates information through movement. This generativity of the body provides the instrument for the expression of practical knowledge and beliefs as well as cultural norms for conduct through actions.

#### Mimesis as Body Metaphor

The mimetic capacity to intentionally communicate through behavior provides a medium for social sharing of internal knowledge and experience. Mimesis produces symbols and meaning through metaphors expressed through enactment, a mapping of the actions of the body onto an imagined reality. This analogy derived from the body’s movements and their intrinsic meanings provides a general expressive medium for communicating to others information about our inner states and past experiences, as well as future plans. Mimesis is a conscious production of meaning through use of behavior, gesture, imitation and emotion for enactment; this mapping of body actions onto an imagined context is a process of analogical transfer that exploits the body’s innate schemas which are the template for all knowing (see Winkelman, 2010a). This lived body provides the basis for our resonance with others, a sensorimotor self with visceral responses that derives understandings of others and the meanings expressed in mimesis through the operation of the mirror neurons.

#### Mimetic Consciousness as Empathy

Donald suggested that mimesis, by focusing attention on the body’s movements, produces a physical self-consciousness that enhances awareness of self. Mimesis enhances personal and social consciousness by providing a mechanism for sharing the representations produced within our own brain and in other brains. Girard (1987) proposed mimesis provides the ability to perceive others as a “mimetic double,” an alter ego or double inside oneself that stimulates self-reflection and consciousness. This awareness made possible by mimesis and imitation provided shared information that created a foundation for culture, customs, rituals, communicative gestures, and learned skilled behaviors and shared group consciousness and culture.

Imitation is the foundation of affective responses underlying human relational motivations, social attachments and empathy (Garrels, 2005). Mimesis provides the basis for empathy through shared and reciprocal social experiences. Because of the common neuronal pathways activated by engaging, observing and even imagining a specific action, mimesis provides a basis for empathic resonance with and understandings of others. Mimetic mechanisms provide a direct means for communication of internal mental states and the meaning of behavior, a foundational basis for empathy by communicating the meaning to actions. The meanings of our actions are manifested in the intentions and goals exhibited in our interactions with the physical and social world and in the desires and beliefs they manifest. This combined mimetic expression of inner and external realities, the body and the social, provides the ability to acquire insight into others’ thoughts through the representations produced within the body.

The innate bases of mimesis and imitation enable their operation from the first days of infancy in the development of affective relations with caretakers. Imitation is a fundamental mechanism for the development of the individual’s mind and the expression of complex representations, including theory of mind (Garrels, 2011). Imitation and mirror neurons provide the basis for the identity of self and others, with the identity experienced through understanding the minds of others. Imitation continues to play a fundamental role across life, functioning even through adulthood as a basic organizing principle in human interpersonal behavior (Garrels, 2005). This mimetic system is the basis of emotional expression and the human capacity to interpret complex social situations and attribute meaning to others’ behaviors.

### Mimesis in Shamanic Evolution

Social primates develop a wide variety of ritualized behaviors to enhance trust, promote harmony and intensify social bonds among members of the group. Such group bonding ritualizations found among other primates indicates they were preadaptations expanded across human evolution. A significant effect of ritual is enabling an approximation of individuals, a reduction of natural suspicion, defensiveness and hostility (Rossano, 2009). Rossano characterizes the central rituals of traditional societies as involving expression of dangerous emotions that must be controlled, as well as the control of others’ reactions (inhibiting a mimetic emotional response). The highly emotional rituals demand considerable inhibitory control for successful ritual participation, a suppression of pre-potent emotional responses, especially anger and aggression. The ability of our ancestors to inhibit aggression and defensiveness allowed for ritual to expand feelings of trust and the formation of long-term alliances that enhanced survival.

The adaptiveness of ritual lies in the creation of a sense of a common group bond and identity that helps to overcome the natural tendency toward ethnocentrism and maintenance of in-group boundaries that excludes outsiders. Shamanic rituals helped forge commonality through the mimetic enactments and the ritual alteration of consciousness that produced a sense of unity with others. Ritual behaviors were able to enhance social integration because of their intrinsic ability to inhibit innate aggressive tendencies and inhibit defensive behaviors, thereby producing interpersonal conditions that facilitate social bonding (Rossano, 2009). This inhibition provides fitness advantages, enhancing social bonding mechanisms, increasing status and access to resources, and providing psychophysiological benefits from eliciting endogenous healing responses.

#### Rituals and Costly Displays

The mimetic capacity greatly expanded the expressive capacities of ritual, typified in costly, conspicuous or extravagant displays. These displays are considered costly energetic activities that appear to have individual costs but that enhance group solidarity as hard to fake signals that provide credible and reliable evidence of the individual’s fitness and group commitment (Wood, 2017). This concept of costly displays illuminates how hominid behaviors exhibited in the maximal displays expanded across hominin evolution through drumming, singing, and dancing. Drumming is costly display that is a widespread mammalian adaptation (Randall, 2001). Foot-drumming both alerts members of one’s group and functions as a mechanism for inter-species communication as a manifestation of excessive fitness, of heightened vigilance and a readiness to act. The short bursts of drumming characteristic of chimpanzees (a few seconds in length) and short bipedal charges are dramatically expanded in shamanism in overnight drumming and dancing which has a much more energy costly profile, illustrating that these abilities underwent significant expansion across hominin evolution.

The significance of costly displays in cultural evolution is in their ability to enhance success in competition between groups through practices that contribute to stronger group commitments that increase within-group cooperation. Shamanic ritual performances demonstrate such commitment and excessive fitness in prolonged and extensive energy expenditures of over-night drumming, dancing and singing that requires extensive exertion. Selection for the capacity to drum and dance all night likely also reflects its ability to deter predators (Winkelman, 2010a; Rossano, 2015).

The aversive experiences associated with shamanism (initiations through induced pain, food and water deprivation, exposure to temperature extremes, hours of dancing, drumming and singing) require inhibition of normal impulses and visibly demonstrate excessive fitness and commitment. The ability to endure these painful and exhausting rituals both signals social commitment and elicits such commitment (Rossano, 2015). Costly displays create a tendency in others to nonreflective acceptance and coordinated expression of cooperative tendencies (Shaver et al., 2017). These and other aspects of shamanic ritual activities (i.e., ASC) also repress strategic reasoning and second-guessing of prosocial intentions of others while encouraging cooperation and positive regard in social relations (Winkelman, 2010a). These dynamics which reflect the activation of mimetic mechanisms also elicit endogenous healing responses.

#### Ritual and Endogenous Healing Responses

Homologies between chimpanzee collective displays and shamanic healing rituals indicate communal ritual was the context within which hominins experienced selection for enhanced capabilities related to endogenous healing responses (Rossano, 2006; Rossano, 2007; Winkelman, 2009; Winkelman, 2010b). These pre-adaptations are manifested in chimpanzee aggressive maximal displays and the complementary submission, grooming and reconciliation behaviors that evoke opioid responses.

Rossano (2009), Rossano (2011) proposes that ritual activities provided a significant aspect of the human selective environment because participation in rituals provided enhanced health to those capable of ritual immersion. Ritual was the context for the selection of traits of hypnotizability, a genetic disposition that was fitness-enhancing, by engaging a suggestibility to health-enhancing effects of ASC (McClenon, 2002) and other endogenous healing responses (Winkelman, 2008 Chapters 9 & 10; Winkelman, 2010a Chapter 5). The dominance-submission dynamics of ritual and attachment processes are key to eliciting endogenous healing processes such as hypnotic and placebo responses.

Humphrey (2002) characterized the placebo response as a Darwinian adaptation for addressing threats to health, an emergent property that provided adaptive endogenous healing responses by exapting emotions such as hope that elicit positive responses of the immune system. The most effective mechanism of placebo elicitation is a powerful external authority that reflects high status (age, prestige, and authority) and enhances confidence. In shamanic and other religious healing, the external authority and power of the spirit world is an exaptation of the psychological dynamics of these authority relations to elicit placebo mechanisms. These healing responses include: endogenous opioid responses; parasympathetic responses, evoking relaxation and counteracting stress; enhanced psychoneuroimmunological responses; and hypnotic susceptibility and placebo responses (Winkelman, 2010a).

#### Music as an Endogenous Healing Modality

Music is a modular intelligence and an aspect of mimesis that provides healing mechanisms through its innate ability to affect emotions (Crowe, 2004). Music engages an innate primate biological function for expressing emotions through vocalizations to enhance emotional harmony. Music also induces physiological effects, producing relaxation and stress reduction manifested in lowered blood pressure and cardiac rate and can treat a wide range of health problems because it elicits biologically determined emotional states (Crowe, 2004). Music produces healing through release of repressed emotions, increasing emotional awareness and elevating emotional concerns for their transformation through ritual.

Music manifests emergent properties that communicate basic emotions and transform them into expressions of complex feelings through the coordination of biological, physical, psychological, cognitive and social processes (Cross and Morley, 2009). Consequently, music is a highly effective mechanisms for coordination of emotions, experience and interpersonal relations and contributes to emotional bonding by enhancing coordination and emotional synchrony within the group (Panksepp and Trevarthen, 2009).

This synchrony contributes to enhanced well-being through effects on social emotional systems related to mammalian and group bonding. The mimetic capacities of music and dancing provided technologies that enhanced synchrony and group formation, with coordinated rhythmically repeated motions exemplified in dance extending the mechanisms of social cooperation. By providing a system of coordinated expression of intentions in observable body actions, dance contributed nonverbal communication mechanisms for the creation a shared group consciousness. Mimetic engagements of rhythmic dancing, clapping and chanting provide powerful socialization effects, an engagement of the motor and somatosensory systems in a way that produces powerful affective linkages of the individual with the group and development of social ties and group identity beyond the biological family.

#### The Holistic Imperative

The dramatic displays of the shaman evoke two innate healing principles or processes, the holistic imperative and shamanic projection (Laughlin et al., 1992). The holistic imperative is an innate drive toward health and wholeness, a growth in the structures mediating consciousness, with ASC a “manifestation of the structural drive toward differentiation and reintegration of the neural systems mediating consciousness” (p. 150). The holistic principle also evokes shamanic projection (Laughlin et al., 1992), a form of transference that leads one to unconscious acceptance of control over one’s intentional processes by the shaman (or another advanced adept). The activation of the holistic imperative by ASC then leads one to internalize the projection of a more advanced state manifested by the healer, achieving a higher level of identity, consciousness and integration.

## Psychedelics and the Evolution of Shamanism

The parallels of chimpanzee displays and shamanic rituals reveal a hominid core of collective ritual behaviors that was necessarily the social context within which psychedelics were institutionalized into ancient hominin cultures. Mimesis and costly displays can explain some features of the chimpanzee-shaman ritualization gap (i.e., expansion of the ritual capacities for extended enactment, drumming, dancing, song and music). But many features are not explained by mimesis such as beliefs regarding spirits, animals as personal powers and identity, alterations of consciousness, divination and mythic systems of explanation.

A notable gap between hominid and shamanic ritual involves ASC and spirit world experiences that are at the focus of communal shamanic healing practices. Notably, all three—communal relations, shamanic ASC and healing—are stimulated by psychedelics, exemplified in the concept of entheogens. Cultures around the world have expressed beliefs about the effects of psychedelics that are entheogenic—inherent sources of stimulating internal spiritual experiences (Dobkin de Ríos, 1984; Rätsch, 2005; Winkelman, 2010a). The empirical ability of psychedelics to induce genuine mystical experiences are attested to in double-blind clinical studies, meaning that we have to accept that spiritual experiences occurred when people ingested psilocybin-containing mushrooms.

Psychedelics produce experiences directly related to the chimpanzee-shamanism gap (from Winkelman, 2010a):

- An entheogenic experience of entering a spiritual world;
- a visionary experience of the separation of one’s soul from the body;
- a death-and-rebirth experience producing self-transformation;
- healing, especially through ritual and singing;
- acquiring information through divination;
- an encounter with animal spirits and an experience of transformation into an animal;
- a cosmological system involving an animated nature, animal and plant spirits.

These differences between chimpanzee displays and shamanic ritual indicate a zone of proximal development where psychedelics could have acted as a selective force in evolutionary developments leading to the emergence of shamanism. Shamanic evolution may be explained in part by psychedelics’ effects of stimulating our innate cognitive structures (Winkelman, 2010a; Winkelman, 2013a; Winkelman, 2018) as discussed above in the context of animism and animal powers.

The sociality enhancing effects of psychedelics indicate they could have been key elements in selection for communal ritual healing capacities though Baldwinian processes (where a behavioral adaptation exerts influences for supportive biological adaptations) (see Rossano, 2009; Rossano, 2011; Rossano, 2015). Psychedelics enhance emotional empathy, happiness, trust, and desire for closeness to others (Dolder et al., 2016) and produce positive social-emotional moods and states (Kometer et al., 2012; Preller and Vollenweider, 2016). Overall psychedelic-induced changes in socially oriented personality are manifested in increases in Extraversion, Conscientiousness and Openness (Bouso et al., 2018; Erritzoe et al., 2018).

This psychedelic enhancement of sociality suggests that they exercised influences in selection for those same dispositions in early hominins. The environmentally induced changes on brain function exerted by psychedelics could have become genetically heritable over time as a result of the selective pressure exerted by their physical and psychological effects. The reproductive advantages derived from instrumentalization of psychedelics could have enabled selection for supportive traits that further enhanced exploitation of psychedelic effects (i.e., sociality, visionary experiences, placebo responses, niche construction).

Psychedelic healing was necessarily at first incidental rather than deliberate. This occurred through their effects in enhancing serotonin mediated coping responses, diverse dopamine mediated healing responses, as well as enhancing core aspects of sociality. This treatment became deliberate as hominins learned that psilocybin’s effects on emotions and social relations could be enhanced through ritual in ways that contributed to fitness through their renowned ability to evoke a variety of healing responses (see dos Santos et al., 2016; also see Winkelman and Roberts (2007) and Winkelman and Sessa (2019) for overview).

### ASC: Psychointegration and the Integrative Mode of Consciousness

Cultures worldwide have ASC institutionalized in healing rituals (Winkelman, 1992). These ASC involve stimulation of the dopamine system (see Winkelman, 2013a; Winkelman, 2017b for review), especially through exhausting physical exertion of drumming and dancing for hours which overwhelms temperature-regulation mechanisms, resulting in the release of endogenous opioids (Vaitl et al., 2005). Ritual preparations such as fasting, sexual abstinence and painful ordeals contribute to ASC and the exhaustive dancing and drumming stimulate dopaminergic, serotonergic, endorphin and endocannabinoid systems (Winkelman, 2017b). Features of shamanic activities such as nighttime ceremonies, exposure to pain, emotional manipulations that evoke fear, and song and dance stimulate the endogenous opioid system (Prince, 1982; Winkelman, 2013b; Winkelman, 2019e).

Diverse mechanisms for inducing ASC involve a common biological basis in an *integrative mode of consciousness* (Winkelman, 2010a; Winkelman, 2011). The integrative mode of consciousness is characterized by ascending brain discharge patterns producing integration across levels of the brain, an entrainment of the frontal cortex by highly coherent and synchronized slow-frequency brain wave waves emanating from lower-brain structures. Many practices that typify shamanism produce this overall brain dynamic (Mandell, 1980; Winkelman, 2010a), including: natural drug sources (hallucinogens, amphetamines, stimulants, marijuana, opiates), long-distance running, hunger, thirst, sleep loss, auditory stimuli such as drumming and chanting, sensory deprivation, dream states, meditation, and a variety of psychophysiological imbalances or sensitivities resulting from injury, trauma, disease, or hereditarily transmitted nervous system conditions. Mandel proposed these diverse activities result in loss of serotonin inhibition of hippocampal cells, resulting in increased hippocampal-septal slow-wave EEG activity (alpha, delta and especially theta) that produces hypersynchronous discharges across the hippocampal-septal-reticular-raphe serotonergic circuit. This discharge propagates impulses from the basal areas of the brain into the frontal cortex.

Diverse ASC deactivate central brain control networks—the prefrontal cortex responsible for higher cognitive functions (Dietrich, 2003); and the Default Mode Network (DMN)—which is key to integration of information about self and others (see Winkelman, 2017a for review). Diverse conditions (i.e., endurance running, hypnosis, meditation, psychedelics, dreaming) produce prefrontal cortex deregulation and loss of higher cognitive functions (Dietrich, 2003). The DMN involves a network of structural and functional connections that support meta-cognitive processes activated during an inward focus of attention and introspection and daydreaming. These metacognitive processes are crucial to self-representation, self consciousness, and reflective self-awareness and are activated during mental time travel (projections into the personal past or future) and autobiographical reflection (summarized from Winkelman, 2017a). Psychedelics and other ASC cause disintegration of normal DMN functions, compromising top-down control and producing a fluid brain dynamic with enhanced activation of lower brain input into ascending circuitry to the frontal cortex. This down-regulation permits emergence of information from ancient brain functions.

#### Psychointegration

Winkelman (2010a), Winkelman, (2011) proposed diverse ASC exhibit a common brainwave dynamic of “psychointegration” involving synchronized slow wave brain discharges ascending from lower brain structures and projecting into frontal regions of the brain. Mandell (1980) proposed this neurobiological basis for ASC and their experiential properties as a consequence of effects of diverse agents and activities that activate hypersynchronous discharges in the temporal-lobe limbic and mesolimbic serotonergic pathways and impose brain wave synchronization on the frontal cortex (see Winkelman, 2011; Winkelman, 2013b for discussion). Shamanic practices produce this dynamic of ASC through fasting, exhausting exercise (i.e., dancing and drumming), dream incorporation, the influences of drumming and chanting, and in many cases psychoactive substances.

These general features of psychointegration are typified by the effects of psychedelics on the frontal-subcortical circuits (Vollenweider, 2001) that link the thalamus of the brain stem region with the frontal cortex areas. Psychedelic effects on these cortico-striato-thalamo-cortical feedback loops releases lower brain gating systems, enhancing the flow of information to the frontal areas (Vollenweider and Geyer, 2001). These global dynamics of “psychointegration” enhance integration of information from lower brain processes related to self, emotions, memories, and attachments and integrating them into neocortical areas (see Winkelman, 2010a for review).

#### Shamanic “Dream-Time”

Dreams are central to shamanic ASC, normal physiological processes engaged by the overnight shamanic rituals. Incorporation of dream processes into shamanic ASC was also deliberately produced through dream incubation practices. Because lucid dream experiences are stimulated by activity prior to sleep, ritual drumming and singing likely enhanced lucid dreaming and ASC (see Winkelman, 2010a for review). Shamanism exapted an innate mammalian feature, using an adaptation for learning by producing memory associations during sleep and enhancing information consolidation. Shamanic activities accessed these innate dream processes by using ritual to blend waking consciousness (enhanced by extreme excitation) with dream processes to bring unconscious material into waking consciousness and manage unconscious personality dynamics. Shamanic engagement with dreams used its capacities for virtual scenario construction to engage processes for risk-free consideration of possible options (Brereton, 2000). These visual properties of dreaming typify shamanic ASC that engaged mental images as psychobiological communication processes that integrate unconscious, non-volitional, affective and psycho-physiological information (see Winkelman, 2010a for discussion). This integrates somatic, psychological and cognitive levels through visual images and analogical processes, producing forms of awareness that transcended the embeddedness of bodily consciousness.

#### The Dopaminergic Dynamics of Shamanic ASC

Dopamine is central to shamanic ASC (Previc, 2006; Previc, 2009), activated by a variety of activities and the body’s own endogenous opioids that produce: experiences of positive emotions, euphoria and belongingness; bonding and affiliation; and enhancing coping mechanisms, stress tolerance, and ability to adapt. The ascendance of the dopaminergic and acetylcholine systems produces the parasympathetic collapse that precipitates dreaming, shamanic soul flight and other visionary experiences (Previc, 2006; Previc, 2009). Dopamine is implicated in a variety of visionary experiences (Hobson, 2001; Previc, 2006; Rolland et al., 2014).

Previc (2009) proposes that the role of the dopaminergic system in human cognitive evolution involved facilitation of goal-directed motivation and acquiring distant rewards that require operations in extrapersonal space, a distant imagined reality rather than in immediate personal space. This requires a dopamine-initiated parasympathetic inhibition of extraneous thoughts and sympathetic emotional responses. Dopaminergic-induced effects extend cognitive capacities for exploring distant regions of physical space as mechanisms for exploring the intimate spaces of our mental capacities and personal identities. “[D]opamine is especially well-suited to making connection among stimuli and events and organizing them into mental plans. . . and in “off-line” thinking and strategizing, important components of abstract reasoning” (Previc, 2009: 30). Dopamine influences on extrapersonal responses and context independent cognition are exemplified in “mental time travel,” the ability to experience and think about things other than those in the here and now. “[D]opaminergic activation results in the “triumph” of extrapersonal brain activity over the body systems that anchor our self-concept and our body orientation, as well as a triumph over the more “rational” executive intelligence maintained in the lateral dopaminergic systems” (Previc, 2009; 53). The shamanic personality exhibits the features of highly dopaminergic minds—seeking connections in unseen forces, above average intelligence, goal seeking, confident in their abilities, intense unconstrained aggressive drives, magical ideation about abilities to control others and distant events, and delusions of grandiosity and invincibleness (Previc, 2009).

#### Soul Journeys as Extrapersonal Cognition

Dopamine stimulation of extrapersonal functions are exemplified in shamanic soul flight and out-of-body experiences which exhibit context-independent consciousness perceived as distant from the physical body (Previc, 2009). These processes are also manifested in astral projection and near-death experiences (see Winkelman (2010a) for discussion). The experience of separation of the experiential self from the body is reported in spiritual practices around the world, with the homologous features indicating that it reflects innate psycho-physiological structures. The soul journey engages a visual symbolic capacity focused on self-reference (intrapersonal intelligence) which is integrated with the innate social psychological capacity to take the perspectives of others toward one’s self. The soul journey reflects these capacities for self-awareness and other awareness displayed in the visual symbolic and spatial modalities.

Metzinger (2009) analyzes the phenomenological properties of these experiences as reflecting the separate operation of the innate processes producing the proto-mind and self. This disarticulation of the normal integrated functions of visual, corporeal and self modules provides adaptive functional features producing the separation of cognitive capacities from the physical self-representation. Metzinger proposes these experiences reveal the functional modularization of the brain under conditions of stress that enables the brain’s information-processing systems to redistribute cognitive functions, enabling higher cognitive processes to continue in spite of physical incapacity.

This separation of cognitive functions produces experiences of one’s self as a soul-like entity, a self-awareness and self-modeling that moves beyond the primitive bodily processes and transcends the present moment. This visionary aspect of the shaman’s ASC is a complex synesthesia blending of corporeal and sensory modalities to produce special forms of self-awareness based in the capacity to take the perspectives of other toward one’s self. The soul flight reflects a natural symbol system, based on a neurognostic model derived from the body, a neurological basis for analogical thinking (Winkelman, 2010a).

#### Death and Rebirth as Self-Transformation

A core aspect of shamanic ASC and a psychiatric and entheogenic phenomena is the death and rebirth experience (see Winkelman, 2010a for discussion). The shaman’s death and rebirth initiatory crisis involves the breakdown of forms of identity, a natural process of self-transformation that occurs as a response to extreme stress that produces fragmentation of the ego. During the period of selection and training, the shaman typically undergoes an experience interpreted as their personal death, which may occur spontaneously, or during a vision quest. In either case, the death and rebirth crisis typically involve a sequence of experiences in which animals attack, kill and devour the initiate, and then subsequently reconstruct the initiate, incorporating their qualities as the basis for shamanic power.

The dismemberment experiences are auto symbolic images of breakdown of psychological structures and sense of self, a fragmentation that is followed by a reformulation of the self. Thus the death and rebirth initiatory crisis involves changing the customary programming of self, permitting a transformation of self-reference and an engagement with a new self-development. The death and rebirth phenomena are manifested cross-culturally because they reflect such natural processes of self-transformation that occur under conditions of overwhelming stress and the fragmentation of the ego. Shamans are often driven to their profession by a constant illness that requires they become new kinds of people—healers—in order to overcome their own health problems. Consequently, a “death” of their current identity (as a “normal” but ill person) permits emergence of a new identity as a healer. These reformulations of the self are guided by innate drives toward integration derived from the psychointegration produced by ASC. Holistic imperatives toward psychointegration restructure the ego through integration of repressed and dissociated cognitive structures, alleviating psychosomatic and emotional problems through enhancing self-actualization.

## Discussion: Neurophenomenological Perspectives on the Set and Setting of Shamanism and Psychedelic Ecopsychology

The cross-cultural characteristics of shamanic practices reveal features indicative of our evolved psychology; practices that incorporate these biological bases should enhance psychedelic effects by optimizing set and setting. The homologies of shamanism with key features with chimpanzee maximal displays emphasize the features of the hominid baseline from which shamanism emerged. Furthermore, many core features of shamanism and its ritual set and setting are homologous with typical psychedelic effects, indicating the need to consider shamanic principles as important for structuring contemporary psychedelic therapy.

### Hominin Set and Setting: The Mimetic Suite

Features of shamanic setting have homologies with features exhibited in the chimpanzee maximal display, indicating both the hominid baseline and the deep evolutionary roots of shamanism. These hominid features are: high energy nighttime displays with group unification; enactments of costly displays, the precursors to dancing; dramatic emotional vocalizations (chorusing and singing) by group members; collective hand and foot drumming; and individual and group emotional expression and integration. A significant feature of hominid ritual setting is the collective ritualization that integrates all members of the local community.

Shamanism emerged through the exploitation of the mimetic capacity through ritualized enactment, dancing, singing and vocal imitation (Winkelman, 2010a). The shaman’s enactment of relations with the spirits and journey to the spirit world exploits the symbolic capacities of mimesis to enact in dramatic bodily movements the struggles with the spirits, combined with emotional expressions, including animal imitation. The overall affective semantics is enhanced by shaman’s recounting of the engagement with the spirits and the ASC induced in participants by the rhythmic drumming, chanting and singing.

#### Mimesis and Habitus

The significance of mimesis for psychological well-being is illustrated in Bourdieu’s (1977, 1990) concept of habitus, the ingrained behavioral and social habits, emotional dispositions and habits of perceiving and reacting derived from shared social experiences. The habitus embodies the general cultural dispositions of the physical body acquired through mimesis and involvement with others in social life, especially during early socialization. Through mimesis and internalization, individual learning experiences incorporate habitus into body, shaping the biological, personal and social habits of the person. While habitus has conscious dimensions, it is mostly unconscious, ranging from bodily postures to linguistic and mental habits creating perceptions and emotions. These dispositions are largely acted out unconsciously, manifested in basic assumptions, prejudices, attitudes, moral evaluations that frame one’s habits and relationships with the world, especially personal and cultural identities.

**FIGURE 1:**
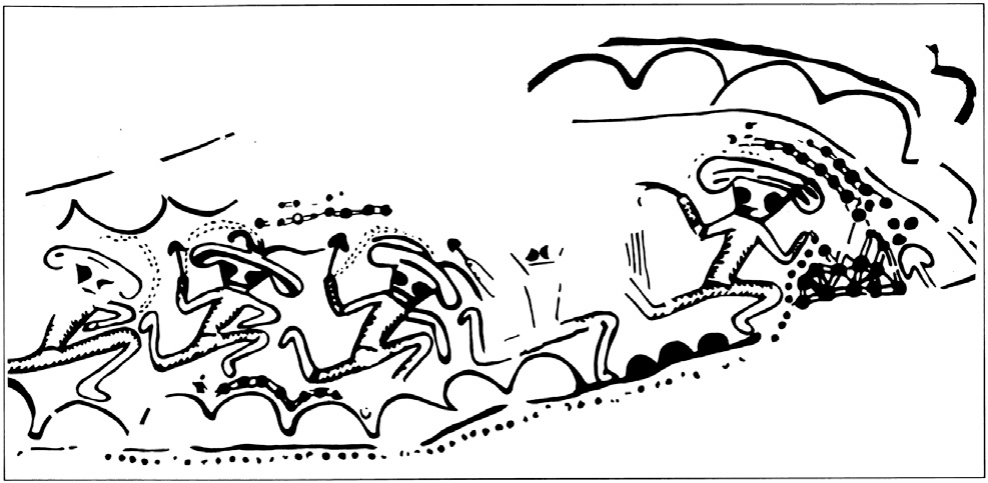
Mushroom Dancers. Relief of a painting in a rockshelter of Tin TazariJt, Tassili, Algeria with dancing figures with a mushroom as a head and a mushroom-like object in the right hand (from Samorini, 1992). Estimated to be 7000-9000 years old. (Photo credit: Giorgio Samorini; used with permission).

This habitus engrains at deep emotional levels not only adaptive behaviors, but also those derived from stressed and pathological social relations. This unconsciously acquired habitus is the basis of one’s orientation to the environment, an embodiment that produces consciousness within the subjectivity derived from the visceral responses of the body. The activities of dance as well as mime and enactment, are the expressions of this habitus. Dancing may be an exceptional modality for both expressing and reprogramming this mimetic-inscribed habitus (see Figure 1). The body-based dynamic of this acquired disposition may be especially amenable to body-based therapies, which should be considered as adjuncts to shamanic and psychedelic therapies (see Razvi and Elffink, 2020).

#### Music

Music, a core feature of shamanic ritual, is part of the mimetic capacity with numerous adaptive psychophysiological effects that both produce psychological and social integration and elicit healing responses. Hominins evolved music and other mimetic modalities because they extended social bonding through the release of endogenous opioids. Music is an innate intelligence that provides a capacity for evoking a range of endogenous healing mechanisms, addressing health problems through eliciting emotional states and concerns and transforming them. The myriad of physiological and therapeutic effects of music make it a natural adjunct for ritual healing, especially psychedelic-induced healing states.

#### Dance

The core role of dance in shamanism and the therapeutic use of dance in cultures around the world reflects its mimetic basis, as well as the diverse mechanisms of action of dance on the body and mind, including contributing to the altering consciousness (Woods, 2009; Winkelman 2010a; Winkelman, 2017b). The role of dance in psychedelic and shamanic set involves its expression of the mimetic body’s socialization and innate impulses. Dance engages our habitus, our reality at the bodily level, the center of our consciousness and identity. This habitualization of behavior acquired through mimesis, can be engaged as a process of active construction through dance, a mimetic re-construction of habitus through engagement of the body in action. Klein (2003) illustrates that the social dimension has an essential role in mimetic learning, a participatory experience involving behavioral relations with others through which one acquires personal dispositions and sensibilities of social behavior.

Woods proposes that dance exercises intrinsic therapeutic effects derived from the elicitation of emotional reactions that facilitate catharsis. ASC produced through dance facilitate experiences of different aspects of the self in personal expressions that liberate repressed emotions in nonverbal expressions of unconscious dynamics. This expression is exemplified in possession phenomena where dance performances manifest participants’ dissociated and repressed desires that are expressed in behavior and utterances attributed to possessing entities. The effects of dance as exercise produces cycles of sympathetic stimulation followed by relaxation (parasympathetic collapse phase), providing an intrinsic ability to both provide energetic stimulation and revitalization, as well as relief of tension and stress. Woods reviews evidence that dances also engage processes similar to hypnotic induction and a release of ego control, which allows for emergence of ancient body-based expressive modalities that promote psychological integration and self-actualization.

#### Healing

Set effects of healing involve elicitation of endogenous healing responses through placebo effects, hypnotic susceptibility and psychointegration of unconscious material. Group ritual dynamics with dramatic authoritative performances enhance hypnotic and placebo responses. The conspicuous displays typical of shamanism should play an important role in conveying these dynamic performances that elicit confidence and contribute to well-being. The collective rituals typified in contemporary raves parallel these collective dynamics of shamanism (Winkelman, 2015b).

### Innate Modular Thought as Set

Shamanic ASC and psychedelics produce a cognitive set by stimulating innate thought processes, particularly intrapersonal and interpersonal awareness and mimetic and musical intelligences. Innate modular thought also structures setting in naturalistic (animal) intelligences and spiritual and existential intelligences that produce spirit experiences and cosmological explanations. Hubbard (2002) characterized shamanic cognition as involving extension of the attributes of human consciousness to the natural world, imbuing it with elements of meaning and intentionality derived from humans’ qualities. Since the stimulation of our innate perceptual structures by psychedelics elevates these ancient innate capacities for detection of agents, an natural aspect of the psychological set for psychedelic experiences is relations with incorporeal entities. This animistic cosmology of shamanism is a neurotheology (Winkelman, 2004) that is activated by psychedelics and other ASC, a natural world view derived from a biologically structured mode of knowing that expresses our innate animal, social and mental natures. The shaman’s dynamic encounter with the spirit world engages the elevation and integration of these normally unconscious processes wherein occur the personal experiences that are the basis of the shamanic healing.

#### Animism and Animals

Entheogenic (and shamanic) perspectives engage a natural neuropsychological set structuring our relationship to nature, especially in animals that have human-like qualities. Ritual engages our reptilian brain’s behavioral communication systems and our paleomammalian brain’s analogical reasoning and emotional, social, and personal processes. The effects of psychedelics in disabling higher-level integrative centers (prefrontal cortex and DMN) and reduction of serotonergic inhibitory control results in ascendence of these reptilian and paleomammalian brain activities (Winkelman, 2010a; Winkelman, 2017a). The normally repressed sensory, personal and emotional dynamics are transmitted by the ascending networks into the frontal brain and conscious awareness, providing information from the behavioral and emotional functions MacLean (1973, 1990) calls “protomentation” and “emotiomentation,” This information drives the psychointegration exemplified in the effects of psychedelics, a powerful bottom-up brain dynamic informed by the ancient primary processing capacities that produce experiences of self in terms of animals. This is why the use of animal spirits as symbolic systems for self-representation is inherent to shamanic and psychedelic set and setting. They are an intrinsic neurophenomenological ecopsychology in which the qualities of animal species provide natural templates for differentiation of personal identity and incorporation of external models into self-development. Shamanic practices also interpret these animal forms as messages that provide important information as visions.

#### Spirits as Others

Pre-modern psychedelic experience was entheogenic—an experience of spiritual beings activated by the plant and within the self. This encounter with an active social agent exemplifies functions of innate modular cognitive intelligences and cognitive operations that are at the core of shamanism. The animistic worldview reflects an enhanced sociality with a natural world imbued with human qualities. Shamanic relations embody the entheogenic perspective that external entities can enter the person and manifest as spiritual powers, as well as become guides and allies. Shamanism incorporated the influences from these spirits as fundamental to self, producing an ecopsychology based in perceptions of nature as personal, intelligent, spiritual and human-like. Shamanism and entheogenic encounters emphasize a set involving the incorporation of others’ as self, reflecting an extension of the social mind’s inference system beyond normal limitations because spirits are presumed to have fuller access to strategic information.

### ASC as Shamanic Set and Setting

Shamanic ASC have both personal and collective dimensions, emphasizing their role as both set and setting. The shamanic set for ritual involved various methods of inducing ASC, beginning with preliminary fasting or dieting, sexual abstinence and sleep deprivation, conditions that produce biological and psychophysiological changes (Winkelman, 2013b). These produce ASC and provide extrapharmacological influences that prime and augment effects of psychedelics. This shamanic ritual setting also produced powerful psychophysiological effects through drumming, singing and dancing, which enhanced operation of the endogenous opioid system and neuromodulatory transmitter system functions (Winkelman, 2017b). Consequently, non-pharmacological ASC induction is a fundamental principle of shamanic setting.

Various ASC result in breakdown in higher cognitive processes and self-structures, exemplified in the disintegration of functions of the prefrontal cortex and Default Mode Network, and in the phenomena of soul flight and the death-and-rebirth archetype, a significant set for psychedelic personal transformation. Some shamanic dynamics such as death-and-rebirth and soul flight reflect disarticulation of neurognostic systems. This breakdown of the normal unity of innate modules supporting consciousness produces new forms of consciousness. ASC manifests our innate animistic and social psychology involving the integration of innate thought modules (Winkelman, 2002; Winkelman, 2019d). Shamanic ASC produce a worldview of interconnectedness, reflecting increased global connectivity dynamics that elevate the reptilian behavioral brain and paleomammalian (limbic) emotional brain functions.

#### Dreams and Active Imagination

Shamanic practices include dream incubation as a set preparation prior to rituals and during performance of overnight rituals during normal dream cycles, enhancing the integration of normally unconscious processes into consciousness. Dreams provide a natural extrapharmacological influence for shamanic and psychedelic experiences, an enhanced capacity for accessing and integrating information from the unconscious, especially in visual forms. These normal physiological processes are integrated into conscious awareness by the enhanced features of consciousness produced by ritual (general arousal). The innate processes of dreaming involving enhanced learning and memory integration makes these features important adjuncts for an enhanced capacity to integrate unconscious material into waking consciousness. This integration facilitates the resolution of trauma and conflicts and provides new possibilities in the processes of virtual scenario construction created by the dream functions.

The phantasy imagery present in dreams and produced by psychedelics has a direct relationship to the processes of “active imagination” proposed by Carl Jung (1961). Jung proposed the meditation technique of active imagination as a tool for enhancing access to the unconscious mind and integrating its contents into consciousness. By focusing on material from dreams and the resultant narrative of produced by interpretation, active imagination provides a process for integrating unconscious material into conscious and the ego’s reality. Key tools for creating this access are dreams, as well as active fantasy and imagination that provide access to the contents of unconscious while at the same time maintaining conscious awareness in order to allow for an integration of unconscious dynamics so that these normally hidden dynamics can be addressed. Psychedelics notably do both.

The process of active imagination focuses on the images that emerge from the unconscious mind, exemplified in dreams and other processes such as fantasy that manifest material from subconscious and unconscious processes. By allowing for an emergence of unconscious material with focused consciousness and attention, the active imagination techniques produce psychological integration of the fragmented self and dissociated functions, identities and complexes. A focus on this material, particularly interpretation of the images through the narrative exploration of their meanings, enhances their integration with ego awareness. This parallels the post-session integration activities typical of contemporary psychedelic retreats. These processes not only produce personal integration but also open the potential for the integration of material from the collective unconscious (Jung, 1961).

#### Soul Flight Experiences

The ASCs of shamanism and psychedelics give rise to experiential properties of a spiritual self, embodied in a soul flight, astral body or similar concept of the self apart from the physical body. This epitome of the entheogenic experience, an experience of an in-dwelling spiritual entity, takes various forms in shamanism, including the death-and-rebirth experience involving the disintegration of physical identity as a prelude to natural processes of rebirth and self-transformation.

#### Death and Rebirth

An initiatory experience of death, typified in shamanic traditions as being killed, and devoured by animals, has many manifestations, an archetype of self-disintegration. This disintegrative dynamic undoubtedly underlies the frequent reports of psychedelic users that they are going to die. Shamanic traditions emphasized facing death rather than running, overcoming one’s fears in confronting experiences that could lead to rebirth as a more powerful person.

#### Shamanic Setting as Communal

The set and setting of shamanic practice are spiritual and collective, with the expectation of a transformative healing encounter through intimate contact with spirit powers. Shamanic healing is communal, attended by all in the local group. The shamanic setting extends the social dynamic of ritual, incorporating non-physical social entities in the cognitive orientation of animism and relationality with the powers of nature, animals and the Universe. A setting in nature was typical for many shamanic activities, and an intimate contact with nature was part of most pre-modern settings. Even modern psychedelic users occasionally experience animal contact during their sessions (Luke, 2019). The social setting for shamanic psychedelic use was of two major different formats: a reclusive retreat in nature for shamanic training that may last for weeks to months; and two communal forms. One communal form involved consumption by the local group for an overnight ritual; and the other involving consumption only by the shaman in a communal ritual to enhance diagnostic and healing powers (Winkelman, 2007).

#### Visions and Divination

Shamanic ASC engage the visual system, a key source of shamanic and psychedelic information. A deliberate internal engagement with the visionary images enhances consciousness by accessing intensified visual information and the novel forms of knowledge created by interregional and global brain connectedness. As is the case with supernatural agents, psychedelics and other vision producing processes expand human abilities at scenario building, producing thoughts independent of actual circumstances or perceived limitations. The imagestic scenarios typify unconscious manifestations and the operation of decoupled cognition, with innate inference systems and intelligences operating independent of normal environmental input. Rock and Krippner (2011) characterized shamanic visionary experiences as processes for deciphering images to acquire novel information to enhance group survival by an increased ability to predict future circumstances. Shamans developed ritual processes to produce and integrate these spontaneous images acquired from unconscious processes. This enhanced information connectivity underlies the shamanic use of ASC for divination—access to novel, innate and unconscious knowledge. Seeking such information for personal integration would be a natural setting of shamanism.

#### Metaphysical Intelligence

The adaptive niche construction capacity depended on many evolved brain structures. Two crucial innate intelligences that Gardner added to the original eight in his update (2000) were: Spiritual intelligence, a desire to engage spiritual, noetic and transcendent experiences; and Existential (or metaphysical) intelligence, concerned with cosmic issues, the meaning of life and death and manifested in mythology and pantheons.

Boyer (2017) proposes that these inevitable human tendencies to construct imagined worlds populated with human-like entities is the consequence of our over-developed social intelligence which evolved because of the importance of knowing what other members of our group are thinking and intending to do. Metaphysical intelligence contributes to scenario construction and adaptations for complex social interactions, providing a medium for anticipating possible future responses as a preparation for actually performing them. The so-called fictions of spiritual and existential intelligence serve a functional role in human cognition and social relations, providing simulations for honing social skills and manifested in various forms of human imagination such as daydreaming, active imagination, fantasies and dreams. The ability of psychedelics to stimulate this existential intelligence described by Gardner is renown, manifested in entheogenic cultures’ beliefs regarding the role of these entheogenic deities in the foundation of culture, mythology, agriculture and cosmology.

One powerful quality of psychedelic experiences is the certainty of one’s beliefs that may result. This reflects a modular intelligence, an “existential (or ontological) operator [which] gives a sense of reality to beliefs, regardless of whether they are non-contradictory or contradictory, or counterintuitive, according to neocortical operators” (Ernandes, 2013, p. 33). This limbic emotional operator is fundamental to the strong feelings regarding fundamental truths, whether from scientific, mystical or psychedelic experiences. As MacLean noted: (1973, p. 123): “It seems that the ancient limbic system provides the ingredients for the strong affective feeling or conviction that we attach to our beliefs, regardless of whether they are true or false!" Consequently, an appropriate psychedelic set should consider a healthy dose of skepticism regarding the certainty of experiences that result.

## Conclusion

This transdisciplinary synthesis incorporating insights from anthropology, evolutionary psychology, psychopharmacology, and the clinical sciences and the neurosciences of psychedelics provides an enhanced understanding of the biological bases of shamanic psychedelic practices. Understanding these bases provides insights into our evolved psychology and how to incorporate these features into post-modern best practices approaches to the set and setting for enhancing therapeutic utilization of psychedelics. These can help guide the application of traditional ethnomedicinal wisdom in the interest of psychedelic-mediated improvements in the mental health of post-modern populations.

The recent clinical research on the objective effects of psychedelics provides an empirical basis to make inferences regarding the likely effects of an environmental factor—psychedelic mushrooms—in shaping selection for core features of hominin psychology, sociality and cognition. Psychedelics have deep evolutionary roots with our ecopsychology through action on our nervous system, particularly serotonin and dopamine. Their effects enhance stress responses in producing active modes of adaptation through changing stressor or our responses to them, as well as enhancing environmental and personal awareness and sensitivity to social rituals and their roles in emotional regulation and healing.

Humanity’s evolved psychology involves enhanced capacities that could have resulted from stimulation with these exogenous neurotransmitter analogues and knowledge of these biopsychosocial bases can an optimize psychedelic effects. These biocultural adaptations are manifested in the cross-cultural patterns of shamanistic ritual preparations and ceremonial activities that incorporate a distilled wisdom regarding set and setting features that help enhance the efficacy of the therapeutic experiences by a grounding in an orientation to innate intelligences and their integration and interaction.

The principal interpretative framework provided by this multidisciplinary approach to shamanic and psychedelic set and setting is neurophenomenological (Laughlin et al., 1992), where effects on neurological systems produce specific forms of experience. This is reflected in the innate brain structures stimulated by psychedelics and other ritualized ASC that create the phenomenological experiences of the shamanic cosmology. This phenomenology reflects pharmacological effects on systemic brain functioning that produce a specific type of information, exemplified in visual imagery and the activation of innate modular intelligences. These neurognostic elements and their neurophenomenological dynamics provide the overall context of meaning-making that is an innate set and setting structuring psychedelic experiences and personal psychodynamics.

This neurognostic approach to adapting the structuring of psychedelic experience to our evolved psychology also accommodates principal aspects of pre-modern indigenous practices for optimal utilization of psychedelics. The scientific application of this traditional knowledge is supported by this interdisciplinary synthesis that reveals the biogenetic origins of shamanic ritual activities that prepare the mind-set of users. These involve extrapharmacological effects of ritual practices that enhance the dynamics of psychedelics.
